# Trade-Offs in Relative Limb Length among Peruvian Children: Extending the Thrifty Phenotype Hypothesis to Limb Proportions

**DOI:** 10.1371/journal.pone.0051795

**Published:** 2012-12-13

**Authors:** Emma Pomeroy, Jay T. Stock, Sanja Stanojevic, J. Jaime Miranda, Tim J. Cole, Jonathan C. K. Wells

**Affiliations:** 1 Division of Biological Anthropology, Department of Archaeology and Anthropology, University of Cambridge, Cambridge, United Kingdom; 2 Child Health Evaluative Sciences, The Hospital for Sick Children, Toronto, Ontario, Canada; 3 CRONICAS Centre of Excellence in Chronic Diseases and Department of Medicine, School of Medicine, Universidad Peruana Cayetano Heredia, Lima, Peru; 4 Childhood Nutrition Research Centre, Institute of Child Health, University College London, London, United Kingdom; University of Missouri, United States of America

## Abstract

**Background and Methods:**

Both the concept of ‘brain-sparing’ growth and associations between relative lower limb length, childhood environment and adult disease risk are well established. Furthermore, tibia length is suggested to be particularly plastic under conditions of environmental stress. The mechanisms responsible are uncertain, but three hypotheses may be relevant. The ‘thrifty phenotype’ assumes that some components of growth are selectively sacrificed to preserve more critical outcomes, like the brain. The ‘distal blood flow’ hypothesis assumes that blood nutrients decline with distance from the heart, and hence may affect limbs in relation to basic body geometry. Temperature adaptation predicts a gradient of decreased size along the limbs reflecting decreasing tissue temperature/blood flow. We examined these questions by comparing the size of body segments among Peruvian children born and raised in differentially stressful environments. In a cross-sectional sample of children aged 6 months to 14 years (n = 447) we measured head circumference, head-trunk height, total upper and lower limb lengths, and zeugopod (ulna and tibia) and autopod (hand and foot) lengths.

**Results:**

Highland children (exposed to greater stress) had significantly shorter limbs and zeugopod and autopod elements than lowland children, while differences in head-trunk height were smaller. Zeugopod elements appeared most sensitive to environmental conditions, as they were relatively shorter among highland children than their respective autopod elements.

**Discussion:**

The results suggest that functional traits (hand, foot, and head) may be partially protected at the expense of the tibia and ulna. The results do not fit the predictions of the distal blood flow and temperature adaptation models as explanations for relative limb segment growth under stress conditions. Rather, our data support the extension of the thrifty phenotype hypothesis to limb growth, and suggest that certain elements of limb growth may be sacrificed under tough conditions to buffer more functional traits.

## Introduction

The environments occupied by contemporary human populations are characterised by a wide variety of ecological stresses, including thermal load, altitude, dietary niche and disease load. Developmental plasticity is suggested to be a key mechanism by which organisms adjust to such environmental variability and has been proposed to be a notable characteristic of humans, enabling our species to colonise diverse environments with limited technological assistance [1]. Nonetheless, variable growth plasticity has been observed in many species. For example, brain-sparing growth, whereby the growth of other organs or tissues is sacrificed to protect the brain under conditions of environmental stress, is a widely-accepted phenomenon in mammals in response to a range of stressors including poor nutrition and hypoxia [2]–[5].

There is also evidence that total lower limb length is more plastic than head-trunk height (commonly measured as sitting height) among humans [6]–[8], but the reasons for this are unclear. This heterogeneity in plasticity is relevant to health: a greater risk of various chronic diseases, including cardiovascular disease, hypertension, diabetes, obesity, liver dysfunction and dementia is associated with shorter stature and with absolutely and relatively shorter lower limbs, but is not associated with head-trunk height [9]–[11] (though see [12]–[14]).

While only relative lower limb length is typically considered, limb segments (stylopod: humerus or femur; zeugopod: ulna/radius or tibia/fibula; autopod: hand or foot) may also be differentially sensitive to stress-related growth disruption. Previous studies of human populations suggest that zeugopod elements may be more sensitive than their respective stylopod elements, and the tibia may be more sensitive than the ulna/radius. Within the limb, it has been demonstrated that zeugopod elements are more variable than stylopod elements, and the tibia more than the radius [15]–[16]. Perhaps more pertinently for the present study, which is concerned with relative limb segment variation in relation to stress exposure, greater positive allometry relative to stature has been demonstrated in lower than upper limb length, and strongest of all in tibia length for archaeological and 19^th^–20^th^ century skeletal samples [16]–[17]. This does not necessarily equate to greater environmental sensitivity, although this interpretation has been proposed [17]. Studies of modern populations also suggest that total lower limb length may be more sensitive than total upper limb length [18]–[21], while the relative sensitivities of autopod lengths are less well known (although see [11]). Therefore the patterning of environmental sensitivity in different limb segments remains to be thoroughly documented, and has implications for understanding the nature of adaptive growth trade-offs under stress conditions.

The reasons for the variable environmental sensitivity of different anatomical regions are also unclear. Three hypotheses may be relevant to explaining this heterogeneity in growth plasticity, and so may help to further our understanding of the underlying mechanisms and the basis of associations between body proportions and disease risk.

First, the thrifty phenotype hypothesis [22] suggests that individuals exposed to environmental stress pre- or post-natally sacrifice the growth of certain organs or tissues (e.g. pancreas, liver and skeletal muscle [23]–[24]) to protect organs whose function would be more detrimentally influenced by impaired growth (e.g. heart, brain). While it has been suggested that trade-offs in lower limb length and head-trunk height are a stress response [25]–[26], other limb proportions have not been explicitly investigated in terms of this hypothesis. As stated above, it is widely accepted that head (brain) size is most protected from the growth-reducing effects of stress, but head-trunk height may be more protected than limb length because the former houses major organs whose function may be impaired by reduced growth [25]. Within the limbs, the autopod (hands and feet) may be more protected from stress exposure than the zeugopod elements to maintain the more specialised functions of the former. There is currently no direct evidence for this suggestion or for the extension of the thrifty phenotype to autopod size. However, it has been proposed that the need for the hand to perform fine manipulation and for the foot to interact effectively with the ground during locomotion could explain why their growth should be more strongly canalised, as stronger natural selection on autopod elements might be expected to ensure their effective function in behaviours that are fundamental to survival [27]–[29].

Second, the distal blood flow hypothesis was proposed in order to explain the greater reduction in tibia length than head, trunk or upper limb dimensions in foetuses exposed to prenatal hypoxia through maternal smoking during pregnancy. It assumes that the tibia is more affected by foetal hypoxia than other limb long bones, head-trunk height, upper limb length or head size, because the tibia is the last among these regions to receive oxygenated blood due to the nature of the foetal circulation [19], [30]–[31]. Relative hand and foot lengths were not specifically considered in this model as originally formulated. This hypothesis could potentially be extended to include other nutrients and postnatal growth patterns, and would predict a proximo-distal increase in environmental sensitivity along the limb, with the lower limb being affected more than the upper. However, empirical evidence for this model is currently lacking [25].

A third possibility that must be considered is adaptation to cold, since the impact of temperature adaptation on body size and proportions has long been recognised in the form of Bergmann’s and Allen’s ‘rules’ [32]–[33]. Allen’s rule predicts relatively shorter extremities (e.g. limbs) in colder environments, and has been shown to apply to a wide range of species including humans [34]–[35]. However the mechanisms underlying such adaptations are not well understood. Cold-induced vasoconstriction may play a role in reducing nutrient delivery to the extremities, but experimental work has also shown that cold temperatures directly affect growth rates by modulating cartilage proliferation [36]–[37]. If temperature influences body proportions, a gradient of reduced growth along each limb would be expected, in a similar manner to that predicted under distal blood flow model. Thus these models are mainly distinguished by details of their underlying mechanism: temperature adaptation involves a more active mechanism of reduced nutrient delivery through decreased peripheral blood flow, in addition to any direct effects of temperature itself, while the distal blood flow model invokes a more passive mechanism of resource depletion with increasing distance from the heart.

**Figure 1:**
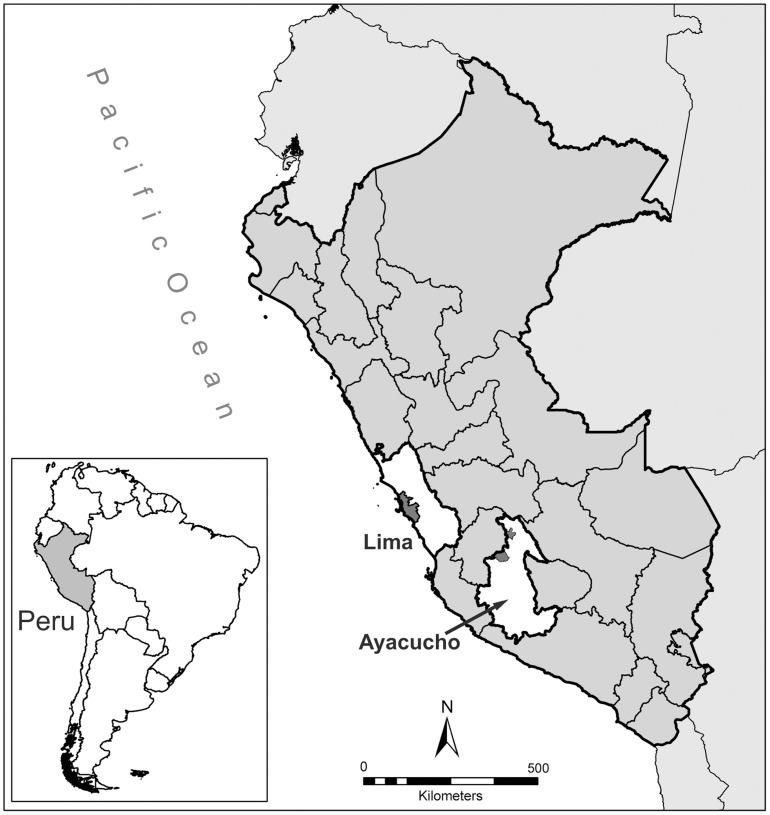
Map of Peru showing location of study sites. Lima and Ayacucho Regions illustrated in white. Dark grey areas illustrate Lima metropolitan area and Districts of Vinchos (south) and Santillana (north) in Ayacucho.

The first aim of this study was to investigate heterogeneity in the sensitivity of different regions of the body to childhood conditions through the comparison of two populations with contrasting burdens of ecological and environmental stress. We analysed total upper and lower limb lengths, zeugopod and autopod lengths; head-trunk height; and head circumference in highland (n = 200) and lowland (n = 247) Peruvian children aged 6 months to 14 years, using cross-sectional anthropometric data (Figure 1). In Peru, life in the Andean highlands is associated with a range of environmental challenges including hypoxia, poorer nutrition and healthcare access, poverty, and cold exposure [38]–[39]. Lowland children also experience stress, particularly poverty, although the overall stress load is markedly greater in rural highland communities: multi-stress highland environments result in higher rates of childhood stunting and wasting compared with the lowlands [40]. The comparison of Peruvian highland and lowland communities therefore offers an interesting context in which to explore the role of developmental plasticity in accommodating exposure to stress.

Given that the phenomenon of brain-sparing growth has been widely demonstrated and accepted, we explored site differences in the lengths of the limbs, limb segments and head-trunk height after adjusting out the variance due to variation in head circumference. This approach highlights variation in the size of distinct regions of the body over and above that in head size, which is most extensively but incompletely [41] protected from growth restriction. Data were analysed using ANCOVA performed in SPSS 17.0. Site differences in head circumference z score were tested using ANCOVA, with sex and age included in the model. A separate ANCOVA analysis was undertaken for each anthropometric measure (dependent variables: total upper limb length, total lower limb length, head-trunk height, and zeugopod and autopod lengths for the upper and lower limbs). Head circumference and age were entered as covariates, and site (highland or lowland) and sex were entered as fixed factors. Tests for significant differences in anthropometry between sites were performed using the Least Significant Difference (LSD) test on the estimated marginal means from which variance due to age and head circumference had been removed.

Anthropometric variables were converted to z scores prior to analysis using the LMS method (see Materials and Methods). Z scores are widely used in paediatric research in order to adjust data for age effects so that characteristics can be compared across a range of ages. They represent the position of an individual within the frequency distribution for a given age in terms of standard deviations from the mean. The use of z scores therefore takes into account variation in body proportions at different ages, as well as differences in the variability of different anthropometric dimensions. All ANCOVA were performed on these z scores.

The second aim of this study was to consider which model (thrifty phenotype, distal blood flow or temperature adaptation) the results do not support. If the thrifty phenotype hypothesis offered the most appropriate explanation, it was predicted that the smallest population differences would be observed in head circumference in line with the brain-sparing model, and that when variance due to variation in head circumference had been adjusted out, head-trunk height and autopod lengths would show smaller population differences than total limb or zeugopod lengths, since the growth of the former is hypothesised to be relatively more protected from stress exposure to maintain function. In contrast, if the distal blood flow model best accounted for the patterns observed, a gradient of increasing population difference in limb or limb segment length was predicted, and it was expected that the upper limb would show smaller size differences between samples than the lower limb. Similarly, temperature adaptation would predict an increase in population differences from proximal to distal along each limb, though perhaps without any difference between upper and lower limb. Under all models, adjusting out the variance due to head circumference z score, head-trunk height would be predicted to show the smallest differences between samples, though the three theories would invoke different physiological mechanisms.

## Results

Tables S1,S2,S3,S4 give summary statistics for anthropometric measures by age group and differences in raw measurements, percentages and z scores. Lowland children are larger than highland children in all dimensions, with absolute differences increasing from the youngest age group until 10 years. Head circumference shows the smallest population differences as expected, although ANCOVA results indicate a significantly greater head circumference by 0.852 z scores in lowland children compared with highland children (p<0.001). This finding is consistent with evidence that the brain-sparing effect may be incomplete [41] and previous reports of reduced head circumference at altitude [42]–[43].

The ANCOVA results confirm that even when adjusting out variance due to head circumference z score and age, significant differences remain between groups in head-trunk height and limb length z scores (Table 1). Site differences in mean length z scores, adjusting out the variance due to head circumference z score and age, are significant (p<0.001 in each case).

**Table 1:** Results of ANCOVA analyses for site differences in length z scores, including head circumference z score and age as covariates in the model.

Anthropometry Z score	Lowland-highland difference in estimated marginal mean	p value for term in ANCOVA				Adjusted r^2^
		age	sex	Head circ. z score	Site	
Head-trunk height	0.67	0.2	0.9	**<0.001**	**<0.001**	0.414
Total upper limb length	1.23	0.3	0.7	**<0.001**	**<0.001**	0.567
Total lower limb length	1.21	0.07	0.6	**<0.001**	**<0.001**	0.494
Ulna length	1.27	0.4	0.8	**<0.001**	**<0.001**	0.578
Tibia length	1.33	0.3	0.8	**<0.001**	**<0.001**	0.586
Hand length	1.07	0.8	0.8	**<0.001**	**<0.001**	0.468
Foot length	1.00	0.5	1.0	**<0.001**	**<0.001**	0.478

**Bold** text indicates significant p values for terms in the model. Head circ. = head circumference.

**Figure 2:**
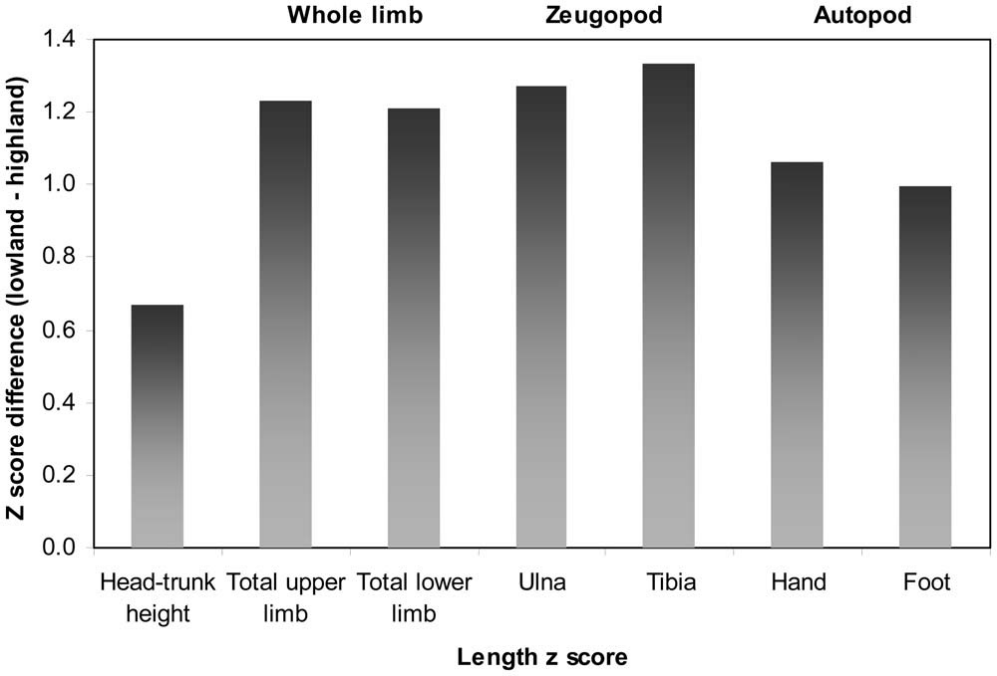
Site differences in estimated marginal means for length z scores from the ANCOVA analyses, demonstrating greater contrasts in total limb and zeugopod lengths, intermediate differences in autopod lengths, and the smallest site differences in head-trunk height.

Figure 2 illustrates the difference in anthropometry z scores between highland and lowland sites, adjusting out the variance due to head circumference z score and age. It demonstrates that the differences in head-trunk height are substantially smaller than in any of the limb measurements. Differences in total upper and lower limb lengths between sites are similar in magnitude. It is notable that site differences in autopod (hand or foot) length are greater than in head-trunk height, but smaller than in total limb or zeugopod lengths.

In each analysis, age and sex terms were not significant, suggesting that the observed patterns do not vary significantly by age. This is partly expected as z scores adjust for age and sex, but if the differences in the relationship between head circumference and z scores had varied with age, this should have been detectable in the analysis. The results therefore indicate that the patterns observed here apply across the range of ages studied (0.5–14 years).

## Discussion

There is clear variation in the magnitude of differences between highland and lowland Peruvian children in the size of distinct regions of the body. Adjusting out the variance due to head circumference z score and age, differences in head-trunk height were markedly smaller than differences in limb or limb segment lengths. In both limbs, site contrasts were smaller in the autopod (hand or foot) than in total limb or zeugopod (ulna and tibia) lengths, and differences in zeugopod length were slightly greater than in total limb length. Observed differences were similar in both the upper and lower limbs and in their homologous segments. These site differences did not show any significant influence of age or sex.

The results do not support the distal blood flow or temperature adaptation models. The similarity in site differences in total limb or limb segment lengths between the upper and lower limb, and the smaller differences in autopod lengths compared with total limb or zeugopod lengths refute the distal blood flow hypothesis, which predicts greater differences in total lower than total upper limb length, and greater differences in autopod than zeugopod length. Differences in ambient temperature are also unlikely to account for the pattern of results. Similar to the distal blood flow hypothesis, a response to cold exposure was predicted to result in a proximo-distal gradient of increasing length reduction from stylopod to autopod, but this was not observed in our data.

Like many previous studies, the results support the phenomenon of brain-sparing growth under conditions of stress. Furthermore, the data suggest the selective protection of certain anatomical regions (primarily head-trunk height and autopod lengths) over others (e.g. zeugopod length), supporting the extension of the thrifty phenotype hypothesis to limb proportions, particularly with reference to autopod size. This hypothesis would suggest that head-trunk height, and to a lesser extent autopod (hand or foot) length, may be protected compared with zeugopod or total limb lengths for functional reasons, although this interpretation remains hypothetical until we are able to understand better the mechanisms underlying the patterns observed here.

We suggest that site differences in body proportions reflect differential overall exposure to environmental stress. The highland population is more deprived in terms of socioeconomic position and access to education and healthcare, and suffers greater exposure to poor nutrition, hypoxia and cold than the lowland population. Elucidating the relative influences of these various stressors was not possible in the current dataset due to the high inter-correlation between potential explanatory factors. This would require comparison of sites with greater overlap in their socioeconomic, nutritional and health characteristics. Furthermore, environmental stressors like hypoxia and nutrition may have different effects on growth and interact to affect final body size and proportions [44], adding complexity to the relationship between stress exposure and differential growth of body components.

It is also considered unlikely that genetic differences between populations explain the results, although this remains to be explicitly tested. Most Peruvians demonstrate predominantly native South American ancestry with a degree of European admixture [45]–[46], but there is relatively little published information on the genetic composition of Peruvian populations. Peru has a long history of European migration beginning in the 16th century, and migration of Chinese and Japanese populations in the 19th and 20th centuries is most notable in coastal towns and cities including the capital Lima [47]. However, ethnic data (which might shed some light on this issue) are not routinely collected in the census [47]–[48], and there is little indication of how ancestry varies regionally. Thorough genetic estimates of admixture are also sparse. Studies using ancestry informative markers (AIMS) have reported levels of Native American markers of over 90% among male residents of Cerro de Pasco in the Peruvian highlands and mean admixture rates of 10% (range 1–64%) in the offspring of highland migrants to Lima [49]–[52]. In Bolivia (a close geographic neighbour to Peru with aspects of shared migration history) women of purported Andean ancestry in La Paz (highlands) and Santa Cruz (lowlands) demonstrate a similarly high level of Native American Ancestry (mean 80%) [53].

A small degree of genetic difference may exist between the study populations, and given known differences in body proportion between some major world populations [54], this could potentially affect the results. Differences have been most clearly documented between populations of African or Australian ancestry compared with those of European or Asian ancestry (Native American groups being considered most similar to the latter) [55]–[57], but overall genetic differences probably account for a small proportion of variation (∼3.6%) in relative sitting height (head-trunk height relative to stature) among world populations [25], [57]. Unfortunately, there is little indication in the literature as to the extent of any genetic effects on the body proportions of Andeans (or any populations for that matter), and correlations between ancestry, socioeconomic position and altitude make the specific impact of genetic ancestry difficult to detect. Comparisons of Andean populations with international standards or reference datasets for body size and proportions encounter similar problems of correlated differences in genetics and socioeconomic conditions between Andean and reference populations.

One study that attempted to determine genetic and altitude effects on relative head-trunk height and lower limb length among Bolivian children was unable to make clear inferences regarding the impact of genetic factors for this reason [55]. While genetic markers (e.g. AIMs) were not measured, among relatively high SES urban children, Spanish surnames were not significantly associated with relative sitting height (proportionally longer head-trunk height compared with lower limb length). A significant association between at least one parent being born in Bolivia and high relatively sitting height was reported, but there was no significant association between body proportions and having both parents born in Bolivia compared with at least on parent born in Bolivia. Another study in northern Chile that used surnames as a proxy for ancestry reported inconsistent results concerning relative sitting height and lower limb length [58]–[60].

In general, the limited data available and migration history suggests that highland populations tend to show a lower level of European and other admixture, although many residents of shanty towns such as Pampas de San Juan de Miraflores are migrants or the recent descendants of migrants from the Andean highlands [48]. The available genetic evidence suggests a degree of admixture is probable in both study populations, but that both populations are likely to be predominantly indigenous South American in origin. While any differences in admixture levels cannot currently be quantified they most likely exert relatively minor influence on the results in light of a likely relatively low level of admixture and the fact that Asian and European populations are not thought to differ greatly in terms of lower limb/trunk proportions [57], although this is an important area for future investigation.

The detailed physiological basis of the relationship between relatively shorter lower limbs and elevated risk of adult chronic disease is still emerging, but it has been suggested that the early growth environment may influence both body proportions and metabolic characteristics which subsequently impact on chronic disease risk. For example, a number of studies suggest that relatively shorter lower limbs act an index of metabolic capacity, and mothers who are shorter, and especially those with relatively shorter lower limbs, show a reduced tolerance of obesity and a greater propensity to develop gestational diabetes [61]–[62].

It should be noted that not all studies report an association between relative total lower limb length and childhood exposure to stress or adult disease risk [63]–[65]. This could reflect differential response rates of pubertal timing and early growth to environmental improvement [66] or in some cases variability in study design, and requires additional investigation. Our study could not consider whether observed differences in body proportions were maintained into adulthood, or whether later catch-up growth may have modified earlier patterns. A prolonged growth period with a slow, reduced adolescent growth spurt has been documented in highland Andean [67]–[69] and other populations [70]–[72] exposed to nutritional and other stress. While differences in pubertal timing and variable body proportions during puberty may have existed between the highland and lowland populations included in this study, the same pattern of site differences was observed across the age groups included, pubertal children comprised only a small proportion of the dataset, and analysis of the data excluding children aged over 9 years did not alter the patterns observed (data not shown).

The present study was unable to address whether differences in body proportions exist from birth, although the results indicated differences from 6 months of age onwards. Data on body proportions in Andean neonates [43], [73] are sparse and provide contradictory results. Previous studies of inter-population differences in relative lower limb length at birth [4], 74–75 have generally suffered from a lack of statistical testing and correction for potentially confounding factors, and it should also be recalled that both maternal nutrition and genetic factors could contribute to any patterns observed among newborns.

The pattern of growth restriction described here could reflect the growth rate of different tissues at the time of stress exposure (i.e. variation in the timing of critical growth periods among organs [76]), or may be an active response with an adaptive, functional basis that prioritises growth in certain anatomical components. Both scenarios were proposed by Barker and colleagues as potential mechanisms underlying the thrifty phenotype hypothesis [22]–[23], but the present study was unable to address this question directly. It is often assumed that total lower limb length is more sensitive to early childhood growth disruption than head-trunk height due to the faster postnatal growth rate of the former [77]–[78]. As adult brain size is achieved by around age 6 years, head circumference may appear to be spared in the current analysis because it is exposed to stress for a shorter period than total limb length and head-trunk height, which grow far more rapidly and for longer after birth [54].

**Figure 3:**
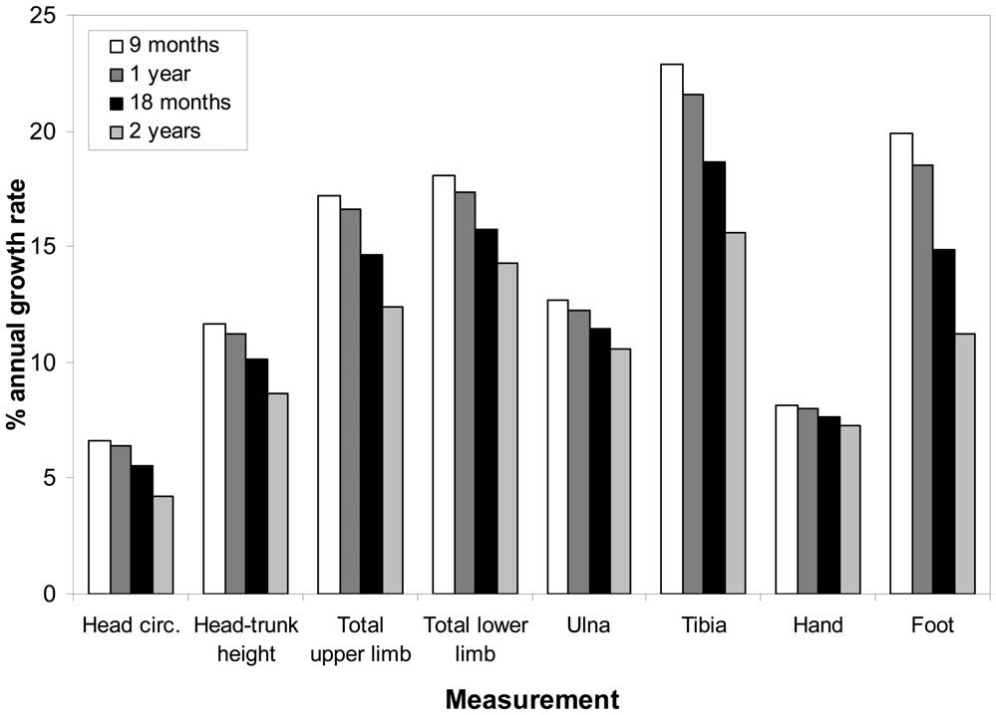
Cross-sectional estimate of percent annual growth rate of limb segments and head-trunk height by age. Growth rates calculated from LMS model median values for 3 months either side of age stated for female participants. ‘% annual growth rate’ refers to the estimated percentage increase in raw measurements over a year based on the median measurement at the start of the 6 month period over which the rate is calculated. Male data demonstrate the same pattern (not shown). Measurements are lengths, apart from ‘head circ.’ which is head circumference.

To provide some initial insight into the relationship between growth rate and sensitivity to stress, a cross-sectional estimate of the annual growth rates of different anatomical regions was calculated from the present dataset using the median measurements at different ages generated by the LMS model. Figure 3 shows that the pattern of estimated growth rates in different body components does not reflect variation in their sensitivity to growth disruption, since the feet are growing fast in infancy but do not appear to be especially sensitive to stress at this time. These data offer preliminary evidence that differential sensitivity of limb segments to environmental stress is not merely a function of relative growth rate. However, alternative explanations are that a relationship between growth rate and sensitivity to growth disruption is established through stress exposure at an earlier age than that included in the present study, or that there are more active mechanisms which alter nutrient provision to different organs under stress conditions.

Studies using animal models suggest that active redirection of blood flow towards the brain and away from the peripheral circulation in response to hypoxia and poor nutrition may account for the brain-sparing effect [5], [79]–[82]. Nonetheless, our results suggest that circulatory effects do not fully explain altered patterns of size in different parts of the body under stress conditions. Detailed investigation of the relationship between the timing of stress exposure and the pattern of altered growth is needed to clarify the relationship between variation in growth rate, the timing of stress exposure, and growth disruption.

The results presented here in relation to head-trunk height and total limb, zeugopod and autopod lengths do not support the distal blood flow or temperature adaptation models. However, the thrifty phenotype hypothesis offers a more ‘ultimate’ explanation for the observed pattern, while the distal blood flow model represents a more ‘proximate’ explanation for observed patterns of body proportions. The two may not be mutually exclusive, particularly for example if the stylopod and zeugopod are compared. The study did not explore differences in stylopod length, and future work might suggest the operation of elements of both models in determining stylopod, zeugopod and autopod proportions.

Whilst our data suggest the differential sensitivity of specific body regions to environmental perturbations of growth, they also indicate that data on body proportions could be used more extensively to assess growth disturbance. The tibia showed the greatest population differences, supporting the use of its length (or knee height) as a particularly sensitive environmental marker [83], and our results also suggest that the ulna is almost equally sensitive to environmental stress. Given their ease of measurement and freedom from the influence of body fatness (in contrast with estimated total lower limb length [44], [83]), tibia and ulna lengths may be particularly informative indicators of childhood environment for future studies.

In summary, our data suggest heterogeneity in the sensitivity of different anatomical regions to stress exposure during growth. Adjusting out the variance due to head circumference, lengths of the zeugopod (ulna and tibia) show greatest differences between sites and so may be most sensitive to stress, while autopod (hand and foot) lengths show smaller differences and so may be relatively protected. Similarly head-trunk height and especially head circumference also appear to be relatively protected from the impacts of stress exposure on growth compared with the limbs. The results do not support the distal blood flow model or temperature adaptation, and therefore support the thrifty phenotype hypothesis, suggesting that this hypothesis could be extended to explain trade-offs in relative limb growth. We suggest ulna length may, like tibia length, offer an additional marker of early-life stress exposure in this population. Further work is required to establish the extent to which different environmental factors affect body proportions and whether our findings extend to other populations.

## Materials and Methods

### Ethics Statement

Participation was voluntary and the study was conducted according to accepted international ethical standards for research involving human subjects (Declaration of Helsinki) [84]. The study was approved by the Institutional Ethics Committee of the Universidad Peruana Cayetano Heredia (Lima, Peru), and by the Ayacucho Region Health Directorate (Dirección Régional de Salud Ayacucho, DIRESA). Written informed consent was obtained from a parent (or legal guardian) by signature or fingerprint where parents were not literate, after the study had been explained in full to them and to the participant in age-appropriate terms. Participants aged 6 years or over also gave their assent, either in written or verbal form where not literate.

### Study Sample

The lowland study population was from the peri-urban community of Pampas de San Juan de Miraflores in Lima (latitude −12.0, longitude −77.0), a well-established but unplanned settlement of generally low socioeconomic status [85]–[86]. The highland populations were from various small, relatively isolated rural agropastoral communities in the Vinchos and Santillana Districts of Ayacucho Region located at 3,100–4,400 m altitude (latitude −13.2, longitude −74.2 for Ayacucho city, Figure 1). A convenience sample of 447 children aged between 6 months and 14 years were selected focusing on children of the following ages in years (range for each group indicated in brackets): 1 (0.5–2), 2 (2–3.5), 4 (3.5–4.5), 6 (5.5–6.5), 8 (7.5–8.5), 10 (9.5–10.5), 14 (13.5–14.5). Date of birth was confirmed from official birth or identification documents, or school records. Only one child per household was included who was born and raised in the study region and was known not to be suffering from any chronic medical condition that might affect their growth (aside from chronic general nutritional problems).

### Methods

Stature and head-trunk height (sitting height) were measured to the nearest mm following standard protocols with participants dressed in light clothes without shoes [87]–[89]. For children below 2 years of age, recumbent length and crown-rump length were measured rather than stature and sitting height respectively using a Rollameter (Raven Equipment Ltd., Dunmow, UK) as is standard practice [90]. In participants over 2 years of age, stature was measured with a Seca Leicester Height Measure following standard procedures [87]–[88]. Total lower limb length was calculated by subtracting head-trunk height from stature (or recumbent length in those aged less than 2 years). Humerus length was measured from the lateral border of the acromion to the inferior extent of the olecranon (elbow flexed at 90 degrees), while ulna length was taken from the olecranon to the head of the styloid process [87]. Humerus and ulna lengths were summed to give total upper limb length. Hand length was measured with palm upwards, fingers and palm fully extended and hand flat. The measurement was made from the level of the ulna styloid to the greatest extension of the middle finger perpendicular to the long axis of the hand [87]. Tibia length was measured from the medial tibial plateau to the end of the medial malleolus and foot length was measured as the maximum length parallel to the long axis of the foot with the participant seated and the foot resting lightly on the ground [91]. All limb segment lengths were measured with large sliding callipers to the nearest mm. Head circumference was measured to the nearest mm with a non-stretch, flexible 10 mm-wide measuring tape (Chasmors, London, UK) following standard procedures [87]–[89]. One observer (EP) took all measurements.

Anthropometric variables were converted to z scores for the combined highland and lowland dataset according by the LMS method [92]–[93] using LMS Chartmaker Light version 2.43 [94]. This converts anthropometric measurements to their relative position in the data distribution for their sex and age, and thus allows pooling of data from children of different ages in analyses. Briefly, the LMS method employs a penalised maximum likelihood method to fit smoothed centile curves to reference data on measurements in relation to a covariate such as age. The method summarises the changing distribution of the data in relation to the covariate by fitting smoothed curves to the skewness (using Box-Cox transformation, λ or L), the median (μ or M) coefficient of variation (σ or S) as cubic splines by non-linear regression, thus producing overall smoothed reference centiles [94]. The extent of smoothing necessary is expressed as equivalent degrees of freedom [92]–[94]. This method has been widely applied in the construction of growth reference centiles for stature, body mass and other measurements [95]–[98].

Differences between the two sites in the size of different anthropometric measurements were tested using ANCOVA of the z scores as described in the Introduction. ANCOVA was selected rather than MANCOVA since the dependent variables violate assumptions regarding multicollinearity required for MANCOVA [99]. This is unsurprising since they are closely related and some measurements are components of others (e.g. zeugopod length is part of total limb length). For the ANCOVA, homogeneity of variances was tested using Levene’s statistic, and found to be significant only for tibia length z score (p = 0.04). A recommended solution to the problem of significant heterogeneity of variances in ANCOVA is to apply a more conservative definition of statistical significance (e.g. p = 0.025 rather than 0.05) in the analyses [99], but as all site differences were significant at p<0.001 this has no effect on the interpretation of the results. We also ensured that data met requirements for a normal distribution, linearity, homogeneity of regression and multicollinearity prior to analysis following Tabachnick and Fidell [99]. Statistical analyses were conducted using SPSS 17.0 for Windows.

## Supporting Information

Table S1 Summary statistics for anthropometric variables age group for lowland (L, n = 247) and highland (H, n = 200) children. Data presented as ‘mean (standard deviation)’, n presented as ‘lowland, highland’.
(DOC)

Table S2 Differences between populations (lowland-highland) in mean raw measurements by age group. Standard error of the difference given in brackets.
(DOC)

Table S3 Percentage differences between populations (lowland-highland) in mean raw measurements by age group.
(DOC)

Table S4 Differences between populations (lowland-highland) in mean z scores by age group. Standard error of the difference given in brackets.
(DOC)
