# Supplementary material for: Trade-Offs in Relative Limb Length among Peruvian Children: Extending the Thrifty Phenotype Hypothesis to Limb Proportions
Source: PLoS One. 2012 Dec 13;7(12):e51795. doi: 10.1371/journal.pone.0051795 (PMC3521697; doi:10.1371/journal.pone.0051795)
Supplement: Table S1 — Summary statistics for anthropometric variables age group for lowland (L, n = 247) and highland (H, n = 200) children. Data presented as ‘mean (standard deviation)’, n presented as ‘lowland, highland’. (DOC) [file pone.0051795.s001.doc]

**Table S1. Summary statistics for anthropometric variables age group for lowland (L, n=247) and highland (H, n=200) children.**

Data presented as ‘mean (standard deviation)’, n presented as ‘lowland, highland’.

| **Age group (years)** |  | **1** | **2** | **4** | **6** | **8** | **10** | **14** |
| --- | --- | --- | --- | --- | --- | --- | --- | --- |
| **Stature (cm)** | **L** | 78.1 (5.69) | 91.0 (4.21) | 101.5 (3.64) | 114.3 (3.67) | 126.1 (5.48) | 136.6 (5.78) | 155.7 (7.46) |
|  | **H** | 72.3 (5.57) | 85.4 (4.85) | 94.8 (4.15) | 104.9 (4.89) | 117.0 (5.93) | 126.4 (5.02) | 146.0 (4.09) |
|  | **n** | 44, 43 | 47, 41 | 30, 29 | 30, 25 | 32, 23 | 29, 28 | 31, 10 |
| **Head-trunk height (cm)** | **L** | 48.5 (3.08) | 54.4 (2.15) | 58.8 (2.53) | 64.3 (2.41) | 69.3 (3.18) | 74.0 (3.25) | 83.7 (4.21) |
|  | **H** | 45.3 (2.88) | 52.3 (2.62) | 56.9 (2.11) | 61.0 (2.30) | 66.0 (2.80) | 70.2 (2.54) | 78.5 (2.22) |
|  | **n** | 44, 42 | 47, 41 | 30, 29 | 30, 25 | 32, 23 | 29, 28 | 31, 10 |
| **Total upper limb**  **length (cm)** | **L** | 26.1 (2.36) | 31.7 (1.61) | 35.4 (1.59) | 40.1 (1.76) | 45.2 (2.19) | 49.2 (2.14) | 56.6 (3.00) |
|  | **H** | 23.9 (1.90) | 28.8 (1.65) | 31.8 (1.55) | 35.9 (1.82) | 40.3 (2.39) | 44.2 (2.06) | 51.89 (1.67) |
|  | **n** | 36, 17 | 42, 36 | 29, 29 | 28, 24 | 32, 22 | 29, 26 | 31, 10 |
| **Ulna length (mm)** | **L** | 116.0 (9.74) | 138.9 (8.36) | 156.3 (7.16) | 176.1 (7.80) | 197.2 (8.77) | 215.1 (11.09) | 249.4 (14.43) |
|  | **H** | 105.1 (8.38) | 125.8 (7.44) | 139.0 (7.45) | 156.1 (8.45) | 174.8 (10.49) | 190.2 (9.49) | 220.2 (7.90) |
|  | **n** | 42, 19 | 44, 36 | 29, 29 | 29, 24 | 32, 22 | 29, 26 | 31, 10 |
| **Hand length (mm)** | **L** | 94.6 (6.42) | 108.3 (5.61) | 118.3 (5.11) | 131.4 (5.13) | 142.1 (6.87) | 155.9 (6.67) | 175.2 (10.35) |
|  | **H** | 89.2 (6.15) | 100.6 (5.27) | 109.4 (5.08) | 120.0 (6.27) | 129.9 (5.83) | 141.4 (6.53) | 159.9 (4.81) |
|  | **n** | 35, 14 | 43, 33 | 29, 29 | 29, 25 | 32, 23 | 29, 26 | 31, 10 |
| **Total lower limb length (cm)** | **L** | 29.6 (3.06) | 36.6 (2.42) | 42.8 (1.85) | 50.0 (2.46) | 56.9 (3.10) | 62.5 (3.10) | 72.0 (4.15) |
|  | **H** | 26.9 (3.10) | 33.1 (2.52) | 37.8 (2.39) | 43.9 (3.00) | 51.0 (3.76) | 56.2 (3.23) | 67.5 (2.56) |
|  | **n** | 44, 42 | 47, 41 | 30, 29 | 30, 25 | 32, 23 | 29, 28 | 31, 10 |
| **Tibia length (mm)** | **L** | 143.5 (15.73) | 179.2 (12.43) | 207.7 (9.11) | 241.8 (12.46) | 276.2 (15.59) | 301.0 (16.20) | 346.8 (20.58) |
|  | **H** | 122.8 (12.86) | 160.3 (11.48) | 180.0 (12.00) | 207.6 (14.02) | 241.9 (17.56) | 262.8 (16.46) | 314.5 (15.20) |
|  | **n** | 42, 27 | 44, 36 | 29, 29 | 29, 25 | 32, 23 | 29, 26 | 30, 10 |
| **Foot length (mm)** | **L** | 122.6 (11.70) | 145.1 (9.08) | 160.2 (7.81) | 179.4 (6.88) | 199.2 (11.16) | 217.5 (11.42) | 237.5 (14.30) |
|  | **H** | 111.9 (8.53) | 134.9 (9.31) | 148.0 (7.67) | 164.4 (9.08) | 180.6 (9.92) | 197.2 (9.13) | 220.0 (9.75) |
|  | **n** | 42, 26 | 44, 37 | 29, 29 | 29, 25 | 32, 23 | 29, 26 | 31, 10 |
| **Head circumference (mm)** | **L** | 45.9 (1.72) | 48.8 (1.14) | 49.5 (1.23) | 50.9 (1.61) | 51.7 (1.59) | 52.9 (1.20) | 54.3 (1.50) |
|  | **H** | 44.8 (1.70) | 47.6 (1.10) | 48.8 (1.14) | 49.9 (1.53) | 50.6 (1.35) | 51.3 (1.22) | 51.9 (0.92) |
|  | **n** | 44, 37 | 45, 37 | 29, 29 | 29, 25 | 32, 23 | 29, 27 | 31, 10 |
