# Supplementary material for: Trade-Offs in Relative Limb Length among Peruvian Children: Extending the Thrifty Phenotype Hypothesis to Limb Proportions
Source: PLoS One. 2012 Dec 13;7(12):e51795. doi: 10.1371/journal.pone.0051795 (PMC3521697; doi:10.1371/journal.pone.0051795)
Supplement: Table S2 — Differences between populations (lowland-highland) in mean raw measurements by age group. Standard error of the difference given in brackets. (DOC) [file pone.0051795.s002.doc]

**Table S2. Differences between populations (lowland-highland) in mean raw measurements by age group.**

Standard error of the difference given in brackets.

| **Measurement (in cm)** | **Age group (yrs)** | | | | | | |
| --- | --- | --- | --- | --- | --- | --- | --- |
|  | **1** | **2** | **4** | **6** | **8** | **10** | **14** |
| Stature | 5.8 (1.58) | 5.6 (1.19) | 6.8 (1.26) | 9.5 (1.63) | 9.1 (2.50) | 10.1 (1.97) | 9.6 (3..01) |
| Head-trunk height | 3.3 (0.66) | 2.1 (0.48) | 1.8 (0.62) | 3.3 (0.65) | 3.2 (0.90) | 3.9 (0.83) | 5.2 (1.25) |
| Total upper limb length | 2.3 (0.60) | 2.8 (0.32) | 3.6 (0.38) | 4.2 (0.47) | 4.9 (0.65) | 5.0 (0.56) | 4.9 (0.82) |
| Ulna length | 1.1 (0.19) | 1.3 (0.14) | 1.7 (0.15) | 2.0 (0.17) | 2.2 (0.20) | 2.5 (0.24) | 2.9 (0.32) |
| Hand length | 0.5 (0.14) | 0.8 (0.09) | 0.9 (0.10) | 1.1 (0.11) | 1.2 (0.14) | 1.4 (0.14) | 1.5 (0.21) |
| Total lower limb length | 2.8 (0.69) | 3.5 (0.51) | 5.0 (0.53) | 6.1 (0.81) | 5.9 (1.16) | 6.3 (0.95) | 4.4 (1.40) |
| Tibia length | 2.1 (0.30) | 1.9 (0.22) | 2.8 (0.22) | 3.4 (0.31) | 3.4 (0.41) | 3.8 (0.41) | 3.2 (0.61) |
| Foot length | 1.1 (0.21) | 1.0 (0.16) | 1.2 (0.17) | 1.5 (0.16) | 1.9 (0.24) | 2.0 (0.24) | 1.7 (0.35) |
| Head circumference | 1.1 (0.34) | 1.2 (0.20) | 0.7 (0.27) | 1.0 (0.39) | 1.0 (0.36) | 1.5 (0.28) | 2.5 (0.35) |
