# Supplementary material for: Trade-Offs in Relative Limb Length among Peruvian Children: Extending the Thrifty Phenotype Hypothesis to Limb Proportions
Source: PLoS One. 2012 Dec 13;7(12):e51795. doi: 10.1371/journal.pone.0051795 (PMC3521697; doi:10.1371/journal.pone.0051795)
Supplement: Table S3 — Percentage differences between populations (lowland-highland) in mean raw measurements by age group. (DOC) [file pone.0051795.s003.doc]

**Table S3. Percentage differences between populations (lowland-highland) in mean raw measurements by age group.**

| **Measurement** | **Age group (yrs)** | | | | | | |
| --- | --- | --- | --- | --- | --- | --- | --- |
|  | **1** | **2** | **4** | **6** | **8** | **10** | **14** |
| Stature | 7.7 | 6.4 | 6.9 | 8.6 | 7.5 | 7.7 | 6.3 |
| Head-trunk height | 7.0 | 4.0 | 3.1 | 5.3 | 4.7 | 5.3 | 6.3 |
| Total upper limb length | 8.0 | 8.1 | 8.9 | 8.9 | 9.0 | 8.4 | 6.8 |
| Ulna length | 8.0 | 7.6 | 8.9 | 8.8 | 8.6 | 8.8 | 8.6 |
| Hand length | 4.6 | 5.5 | 5.8 | 6.6 | 6.4 | 7.0 | 6.5 |
| Total lower limb length | 10.9 | 11.6 | 14.8 | 16.1 | 13.6 | 13.4 | 8.0 |
| Tibia length | 18.4 | 14.2 | 18.8 | 20.5 | 18.3 | 18.8 | 13.3 |
| Foot length | 11.5 | 9.7 | 10.7 | 11.9 | 13.6 | 13.7 | 10.2 |
| Head circumference | 2.3 | 2.5 | 1.5 | 2.1 | 2.0 | 2.9 | 4.6 |
