# Supplementary material for: Trade-Offs in Relative Limb Length among Peruvian Children: Extending the Thrifty Phenotype Hypothesis to Limb Proportions
Source: PLoS One. 2012 Dec 13;7(12):e51795. doi: 10.1371/journal.pone.0051795 (PMC3521697; doi:10.1371/journal.pone.0051795)
Supplement: Table S4 — Differences between populations (lowland-highland) in mean z scores by age group. Standard error of the difference given in brackets. (DOC) [file pone.0051795.s004.doc]

**Table S4. Differences between populations (lowland-highland) in mean z scores by age group.**

Standard error of the difference given in brackets.

| **Z score** | **Age group (yrs)** | | | | | | |
| --- | --- | --- | --- | --- | --- | --- | --- |
|  | **1** | **2** | **4** | **6** | **8** | **10** | **14** |
| Stature | 1.30 (0.11) | 1.47 (0.11) | 1.37 (0.14) | 1.47 (0.15) | 1.36 (0.19) | 1.50 (0.18) | 0.83 (0.19) |
| Head-trunk height | 1.36 (0.16) | 1.12 (0.12) | 0.73 (0.18) | 1.02 (0.17) | 0.95 (0.21) | 1.16 (0.20) | 0.87 (0.21) |
| Total upper limb length | 1.23 (0.16) | 1.72 (0.11) | 1.53 (0.13) | 1.37 (0.15) | 1.56 (0.16) | 1.59 (0.13) | 1.20 (0.19) |
| Ulna length | 1.07 (0.13) | 1.68 (0.11) | 1.70 (0.14) | 1.51 (0.14) | 1.57 (0.14) | 1.64 (0.14) | 1.34 (0.15) |
| Hand length | 0.84 (0.19) | 1.53 (0.13) | 1.38 (0.16) | 1.37 (0.16) | 1.39 (0.17) | 1.59 (0.15) | 1.06 (0.16) |
| Total lower limb length | 1.13 (0.13) | 1.58 (0.11) | 1.70 (0.12) | 1.53 (0.16) | 1.37 (0.16) | 1.38 (0.14) | 0.55 (0.16) |
| Tibia length | 1.43 (0.11) | 1.63 (0.11) | 1.74 (0.12) | 1.62 (0.14) | 1.49 (0.15) | 1.57 (0.13) | 0.86 (0.16) |
| Foot length | 1.07 (0.14) | 1.43 (0.14) | 1.32 (0.17) | 1.27 (0.15) | 1.45 (0.19) | 1.55 (0.16) | 0.67 (0.15) |
| Head circumference | 0.75 (0.16) | 1.14 (0.14) | 0.57 (0.19) | 0.78 (0.25) | 0.67 (0.23) | 1.05 (0.17) | 1.34 (0.22) |
